# Phage tail-like nanostructures affect microbial interactions between *Streptomyces* and fungi

**DOI:** 10.1038/s41598-021-99490-8

**Published:** 2021-10-11

**Authors:** Toshiki Nagakubo, Tatsuya Yamamoto, Shumpei Asamizu, Masanori Toyofuku, Nobuhiko Nomura, Hiroyasu Onaka

**Affiliations:** 1 Graduate School of Agricultural and Life Sciences, Department of Biotechnology, The University of Tokyo, Tokyo, Japan; 2 Department of Life and Environmental Sciences, University of Tsukuba, Tsukuba, Japan; 3 Collaborative Research Institute for Innovative Microbiology, The University of Tokyo, Tokyo, Japan; 4 Microbiology Research Center for Sustainability (MiCS), University of Tsukuba, Tsukuba, Japan

**Keywords:** Microbiology, Bacteria

## Abstract

Extracellular contractile injection systems (eCISs) are structurally similar to headless phages and are versatile nanomachines conserved among diverse classes of bacteria. Herein, *Streptomyces* species, which comprise filamentous Gram-positive bacteria and are ubiquitous in soil, were shown to produce *Streptomyces* phage tail-like particles (SLPs) from eCIS-related genes that are widely conserved among *Streptomyces* species. In some *Streptomyces* species, these eCIS-related genes are regulated by a key regulatory gene, which is essential for *Streptomyces* life cycle and is involved in morphological differentiation and antibiotic production. Deletion mutants of *S. lividans* of the eCIS-related genes appeared phenotypically normal in terms of morphological differentiation and antibiotic production, suggesting that SLPs are involved in other aspects of *Streptomyces* life cycle. Using co-culture method, we found that colonies of SLP-deficient mutants of *S. lividans* were more severely invaded by fungi, including *Saccharomyces cerevisiae* and *Schizosaccharomyces pombe*. In addition, microscopic and transcriptional analyses demonstrated that SLP expression was elevated upon co-culture with the fungi. In contrast, co-culture with *Bacillus subtilis* markedly decreased SLP expression and increased antibiotic production. Our findings demonstrate that in *Streptomyces,* eCIS-related genes affect microbial competition, and the patterns of SLP expression can differ depending on the competitor species.

## Introduction

In their natural habitat, microorganisms are members of densely populated microbial communities that facilitate competitions between neighboring cells for nutrients and niche spaces^1–3^. For example, despite the limited availability of nutrients, soil is one of the most microbially diverse environments, and it is densely populated with different bacterial and fungal species^1–3^. Co-culture model systems using two or more microbial species have been commonly used to investigate competitions between different microorganisms and to identify the factors that influence competitive fitness. These model system have elucidated the functions of many factors, ranging from low-molecular weight compounds to multi-component macromolecular machines^1,4,5^. For example, Gram-negative bacterial type VI secretion systems (T6SSs), which are widely conserved cell envelope-spanning nanomachines, reportedly play important roles in interbacterial competitions by injecting effector proteins into target cells in a cell–cell contact-dependent manner^6^. T6SSs are structurally and functionally homologous to contractile phage tails, indicating the evolutionary relationships of these multi-component machines^7^. Besides T6SSs, another class of phage tail-like nanomachines––extracellular contractile injection systems (eCISs)––have attracted increasing interest owing to their wide distribution among diverse microbes, including Gram-negative and Gram-positive bacteria and archaea^8,9^. Phylogenetic analyses have indicated that eCISs are evolutionarily distant from contractile phage tails and T6SSs^8,9^ despite these sharing many structural and mechanical features^10,11^. Furthermore, it is noteworthy that the currently known eCISs mediate the interkingdom interactions wherein these nanostructures modulate the cellular processes of target eukaryotes^12–15^. However, although eCISs reportedly play important roles in prokaryote–eukaryote interactions, current data on the structures and functions of eCISs are limited to a few Gram-negative bacterial species.

The filamentous bacteria *Streptomyces*, which produces therapeutic antibiotics, is known for the presence of highly conserved eCIS-related gene clusters in 94 out of 116 of their completed genome sequences^9^. These Gram-positive bacteria are ubiquitously found in natural environments such as soil and are well-known for their unique life cycles. First, a spore germinates and the germ tubes grow by apical tip extension. These tubes form a vegetative mycelial network, from which aerial mycelia extend into the air and ultimately form spores. Previous genomic studies have shown that eCIS-related genes in *S. coelicolor* A3(2), an extensively studied model of *Streptomyces*, are regulated by a key regulatory gene, *bldA*, essential for its life cycle^16,17^. Although these observations suggest the biological importance of eCIS-related genes in *Streptomyces*, there has been no direct evidence to support this hypothesis owing to the absence of apparent phenotypic changes in deletion mutants for the eCIS-like genes^18,19^. In addition, the products of these eCIS-related genes in *Streptomyces* have remained unknown.

In this study, we have shown that *Streptomyces* species, including *S. lividans*, produce *Streptomyces* phage tail-like particles (SLPs) from eCIS-related genes. Colonies of *S. lividans* strains that cannot produce these nanostructures are more severely invaded by fungi, suggesting the protective role of producing SLPs in microbial competitions. To the best of our knowledge, this is the first report to show the biological significance of the eCIS-related genes and the macromolecular structures encoded by these genes in Gram-positive bacteria. Further, depending on the competitor microorganisms, patterns of nanostructure expression and antibiotic production in *S. lividans* can differ. Our findings illustrate that *Streptomyces* species employ diverse responses, mediated by both SLPs and secondary metabolites, to microbial competitions.

## Results

### *Streptomyces* species produce SLPs

**Figure 1:**
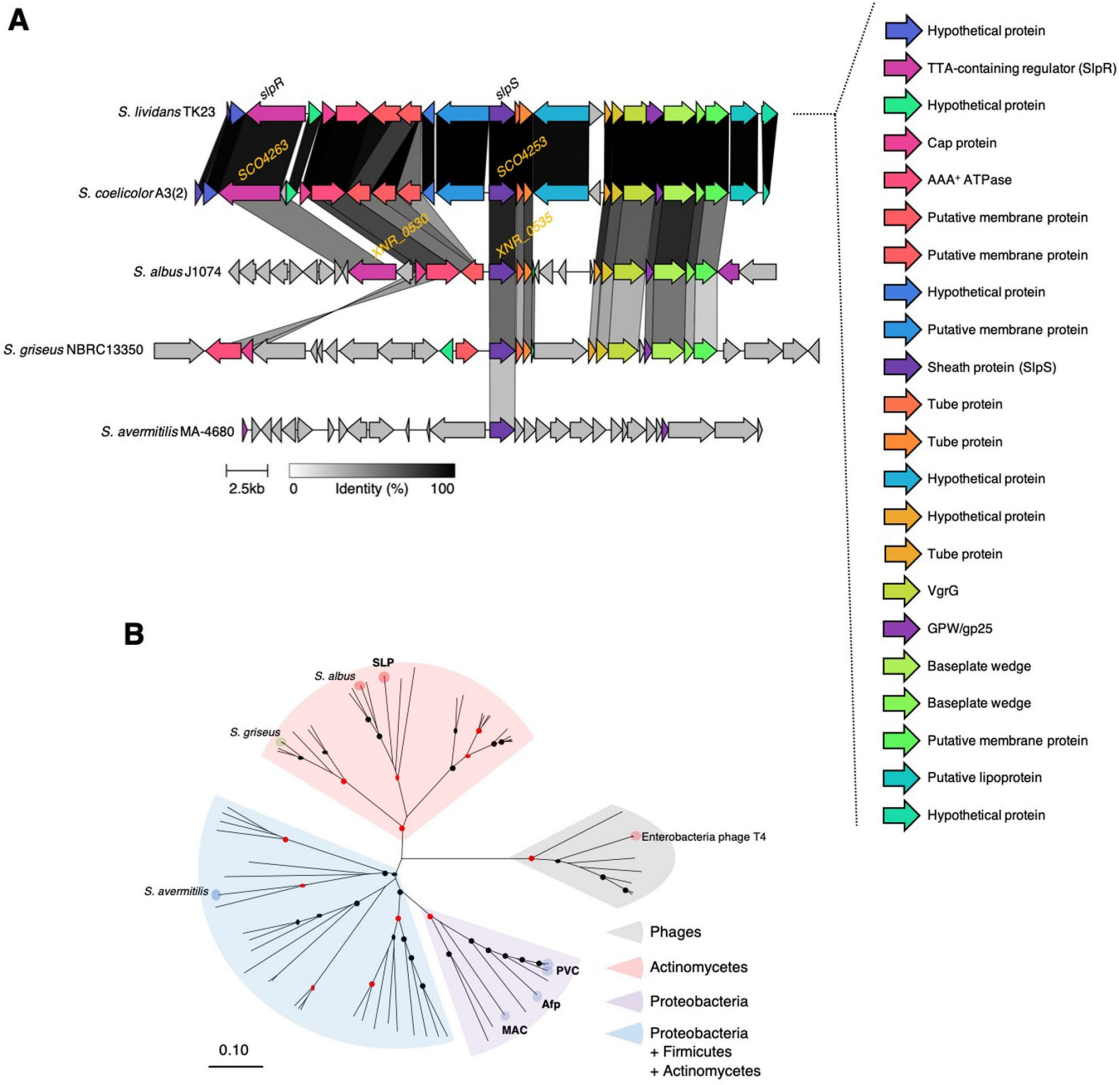
Phylogenetic analysis of eCIS-related genes in *Streptomyces* species. eCIS-related genes are conserved among *Streptomyces*. (**A**) Organizations of eCIS-related genes that are conserved in the model *Streptomyces* species. (**B**) A phylogenetic tree of eCIS and SLP proteins. The tree was constructed based on 72 amino acid sequences of tube proteins using the neighbor-joining method. Red and black circles indicate the clades with the bootstrap values of 100 and 85–99, respectively. The evolutionary distances were computed using the p-distance method and are in the units of the number of amino acid differences per site. Afp, anti-feeding prophage; PVC, *Photorhabdus* virulence cassettes; MAC, metamorphosis-associated contractile structures.

In previous studies, proteomic analyses have shown that phage-related proteins are expressed during the growth of *S. coelicolor* A3(2)^20,21^, although there is no information regarding its biological role and macromolecular structure. The gene cluster encoding these proteins is almost completely conserved in *S. lividans*, a close relative of *S. coelicolor* A3(2) (Fig. 1A). These gene clusters include the homologs of known eCIS genes encoding essential structural proteins (Fig. 1A), suggesting that phage-related genes in *S. coelicolor* and *S. lividans* encode eCIS-like nanostructures. Given that these eCIS-related genes are widely found in other *Streptomyces* species (Fig. 1A), we analyzed the phylogenetic relationships of the eCIS-related genes among actinomycetes and other bacterial classes. Based on a comprehensive annotation of the eCIS genes in the database of extracellular contractile injection systems^9^, we constructed a phylogenetic tree of eCIS-related genes encoding tube proteins^15^ conserved among proteobacteria, firmicutes, and actinobacteria (Fig. 1B). The eCIS-related genes in actinomycetes form a distinct clade that is phylogenetically distant from the functionally characterized eCISs (anti-feeding prophage, *Photorhabdus* virulence cassettes, and metamorphosis-associated contractile structures), which is consistent with that reported in previous studies^8,9^. Notably, the eCIS-related gene of *S. griseus* falls into a subclade within the actinomycetes-specific clade, whereas those of *S. lividans*, *S. coelicolor*, and *S. albus* are members of another subclade (Fig. 1B). In addition, an eCIS-related gene of *S. avermitilis* belongs to a clade that is more closely related to the eCIS genes of proteobacteria and firmicutes, implying that a horizontal gene transfer event might have occurred^8,9^.

**Figure 2:**
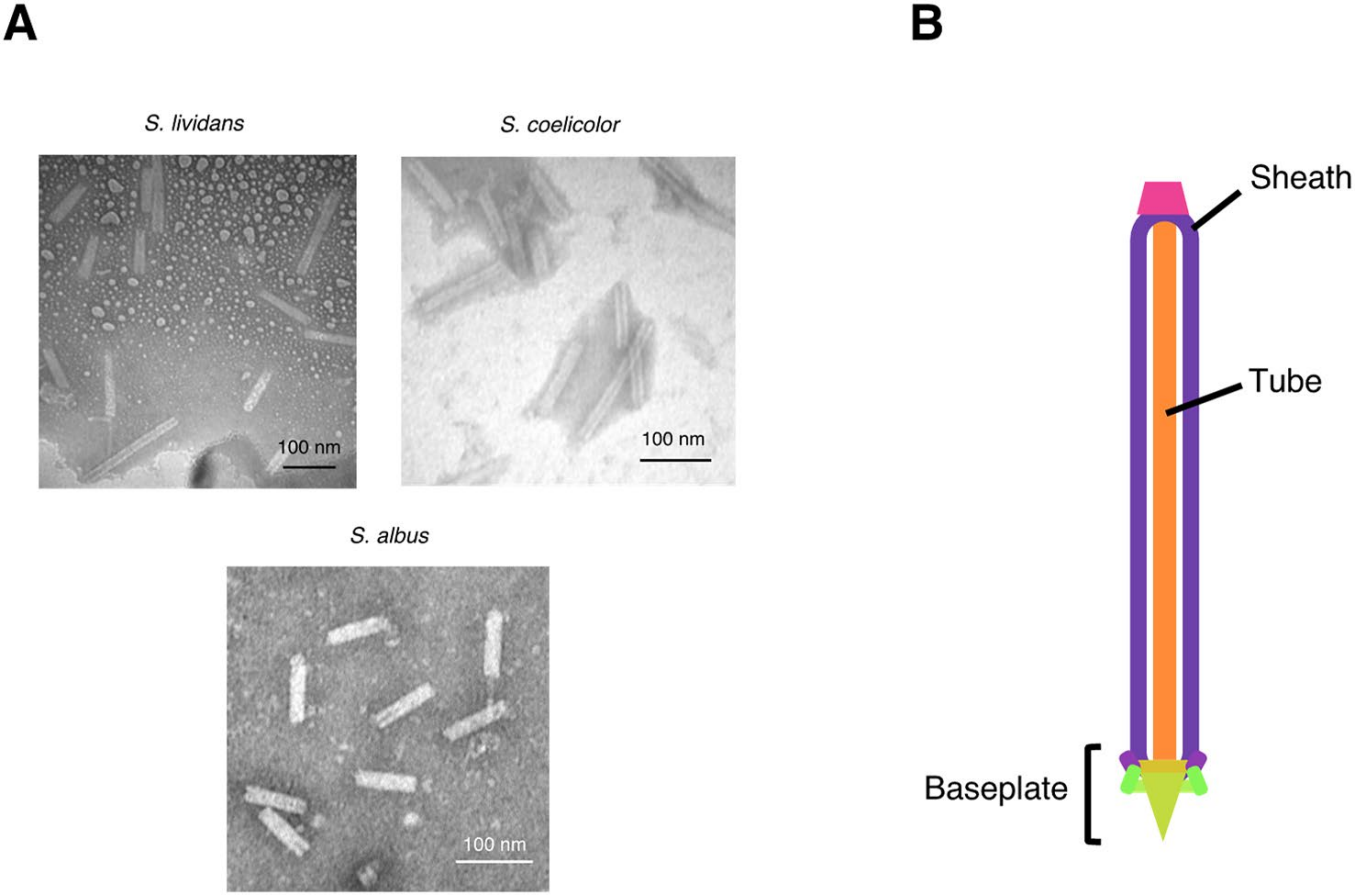
*Streptomyces* species produce eCIS-like nanostructures. eCIS-like nanostructures were found in the mycelial extracts of *Streptomyces* species. (**A**) TEM of the mycelial extracts of *S. lividans*, *S. coelicolor*, and *S. albus*. Scale bars, 100 nm. (**B**) Putative structure of the eCIS-like particle of *S. lividans*. This model is based on Cryo-EM structures of Gram-negative bacterial eCISs (10,11). Each color of the components corresponds to that shown in Fig. 1A.

Next, we investigated whether *Streptomyces* species produce eCIS-like nanostructures. Considering known Gram-negative bacterial eCISs are released into the extracellular milieu via cell lysis^15^, we initially cultivated *S. lividans* and *S. coelicolor* in liquid culture and then ultracentrifuged the culture supernatant. However, we failed to find phage tail-like structures in the resuspended pellets by transmission electron microscopy (TEM), suggesting that these *Streptomyces* species do not release the putative nanostructures into the extracellular milieu under the tested conditions. Given this, we cultivated *S. lividans* and *S. coelicolor* on solid media and performed mild extraction from their mycelia using lysozyme and a detergent. As a result, we found a number of bullet-like nanostructures, which are very similar to known Gram-negative bacterial eCISs^11,15^, in the extracts (Fig. 2A,B). These structures were not observed in the deletion mutant of *S. lividans* for a gene encoding a protein (SLIV_17120, SlpS) that is homologous to Gram-negative bacterial eCIS sheath proteins (Fig. 1A and Supplementary Fig. S1). Because deletion of the sheath proteins was previously shown to abort the assembly processes of eCIS particles^11^, our finding demonstrates that the phage tail-like structures of the tested *Streptomyces* species were synthesized from the conserved eCIS-related genes. Based on these observations, we named the nanostructures “*Streptomyces* phage tail-like particles (SLPs).”

Previous studies have reported that the expression of SLP genes in *S. coelicolor* is regulated by *SCO4263* encoding an LuxR-type transcriptional regulator which is suggested to be a SLP gene cluster-specific regulator^18,19^. *SCO4263* contains the UUA codon, which is very rare in the *Streptomyces* genome that has a high G + C content, in its 5′ region, and its expression is completely dependent on *bldA*^17^, a gene encoding Leu-tRNA^UUA^, which is capable of translating the UUA codon; therefore, SLP genes are regulated by *bldA* in *S. coelicolor*. The *SCO4263* homolog in *S. lividans*, namely *slpR* (Fig. 1A), also contains a UUA codon in its 5′ region, strongly suggesting that *bldA* is a key regulator of SLP expression in *S. lividans* as well as *S. coelicolor*. In fact, SLP structures were not found in deletion mutants for *slpR* and *bldA* of *S. lividans* (Supplementary Fig. S1). This confirmed that *bldA*-dependent regulation of SLP genes is a common mechanism in *S. lividans* and *S. coelicolor*.

To examine whether other *Streptomyces* species produce SLPs, we cultivated three model *Streptomyces* species (*S. albus*, *S. griseus*, and *S. avermitilis*), all of which have eCIS-related genes in their genome (Fig. 1A). Their mycelia grown on solid media were subjected to mild extraction, ultracentrifugation, and TEM, as described above. As a result, we could observe a number of SLP-like nanostructures in the extract of *S. albus*, whereas such structures were not found in the other species (Fig. 2A). These SLP-like nanostructures were not found in the deletion mutants for *XNR0535* and *XNR0530*, *slpS* and *slpR* homologs, respectively, in the eCIS-related gene cluster of *S. albus* (Fig. 1A and Supplementary Fig. S1), suggesting that these nanostructures are the products of the SLP-like genes in *S. albus*. In addition, bioinformatic analysis using PHASTER^22^ suggested the absence of intact or defective phage genes in the *S. albus* genome, further confirming the origin of the SLP-like nanostructures in *S. albus*. Importantly, the *slpR* homolog is present in eCIS-related gene clusters of *S. albus* but absent in those of *S. griseus* and *S. avermitilis* (Fig. 1A), indicating that regulation systems for the expression of latter gene clusters might be different from those of SLPs. This may, in part, explain why SLPs were not found in *S. griseus* and *S. avermitilis* under the tested conditions.

### Loss of SLP genes affects the microbial competition between *Streptomyces lividans* and fungi

**Figure 3:**
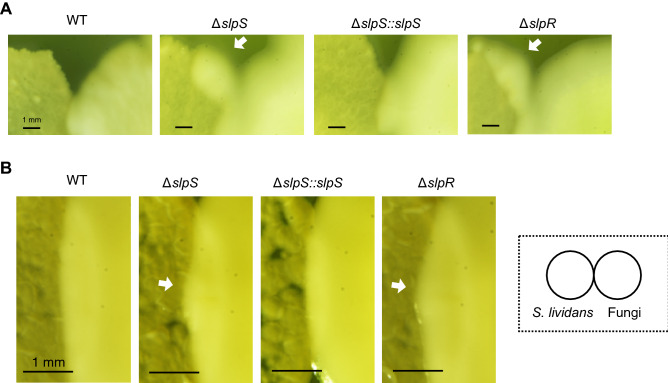
Deletion of SLP genes affects the microbial competition between *S. lividans* and fungi. Microscopic analyses of the microbial competition between *S. lividans* and fungi. (**A**) *S. cerevisiae* and (**B**) *S. pombe* were co-cultured with *S. lividans* on solid YES medium for 7 days. *S. lividans* and fungi were inoculated on the left and the right sides, respectively. White arrows indicate fungal invasion. The *slpS*-complemented strain was constructed by replacing the deleted *slpS* gene with intact *slpS* gene in its native chromosomal locus. In case of co-culture with *S. cerevisiae*, *S. lividans* and the fungus were cultured on a cellophane placed on the solid medium. Scale bars, 1 mm.

Next, we investigated the biological functions of SLPs using *S. lividans* as a model. It has long been known that *bldA*, a key regulator of SLP expression (Supplementary Fig. S1)^17^, is an essential factor in the life cycle of *Streptomyces* species, which encompasses the following developmental stages: spore, vegetative mycelia, and aerial mycelia formation. Many genes essential for these morphological differentiations are regulated by *bldA* and, therefore, the deletion of *bldA* leads to a “naked” phenotype, wherein aerial mycelia and spores are lacking^17^. In addition, *bldA* also acts as a trigger for secondary metabolite production; many gene clusters responsible for secondary metabolite production contain UUA codons, suggesting a pleiotropic role of *bldA* in the *Streptomyces* life cycle^17,23,24^. Given these observations, we first examined the effects of the deletion of the SLP gene on morphological differentiation and secondary metabolite production in *S. lividans*. However, in our experiments, the spore formation rates and the liquid chromatography/mass spectrometry profiles of the extractable metabolites of Δ*slpS* mutant were comparable with those of the TK23 strain. These results support those of the previous study, wherein the absence of any detectable difference in morphological differentiation and secondary metabolite production between M600 strain and SLP-deficient mutant of *S. coelicolor* was reported^18,19^. To further assess the phenotypic consequences of the deletion of SLP genes, we measured the growth of the TK23 *S. lividans* strain and two SLP-deficient strains (Δ*slpS* and Δ*slpR*). This revealed that *slpR* deletion leads to the fast-growing phenotype, whereas *slpS* deletion causes a slight increase in cell weight (Supplementary Fig. 2). Considering that the expression levels of SLP proteins were comparable to those of ribosomal proteins in *S. coelicolor*^21^, we believe that abolishing SLP expression allows the *S. lividans* strain to use more energy and resources for mycelial growth than the SLP-producing strain. In addition, to detect SLP expression and observe its distribution, we constructed an *S. lividans* strain expressing msfGFP-fused SlpS (SlpS-msfGFP). Microscopic analysis revealed that SlpS-msfGFP fluorescence is uniformly distributed in *S. lividans* colonies grown under standard conditions (Supplementary Fig. 3). These results are consistent with those of the previous study showing that SCO4253, a SlpS homolog in *S. coelicolor*, is expressed constantly during growth^18,19^. The abundant and constant expression of structural proteins during the growth phase has also been reported in the T6SSs of Gram-negative bacteria^25^, which may relate to their ecological importance. In addition, it has recently been proposed that some genes, including *bldA*-dependent ones, that are associated with the *Streptomyces* life cycle could mediate interactions between *Streptomyces* species and other organisms^26,27^. These observations led us to hypothesize that producing SLPs may have a significance in the biological interactions that *Streptomyces* species would constantly face in their natural settings. Given that *Streptomyces* species are often found in complex microbial communities in natural environments^28^, we speculated that the deletion of SLP genes lead to phenotypes that are disadvantageous to *Streptomyces* species in microbial competitions that could occur in such environments. For this reason, we co-cultured *S. lividans* and SLP-deficient mutants with various microorganisms and observed the colony morphologies. Among the tested microorganisms, *S. cerevisiae* and *S. pombe* were found to more severely invade the Δ*slpS* mutant colony than the TK23 strain of *S. lividans* (Fig. 3A,B). The *slpS*-complemented strain and Δ*slpR* mutant showed similar phenotypes to the TK23 strain and Δ*slpS* mutant, respectively, in the above competition assay, confirming the involvement of SLPs in the competitive interactions (Fig. 3A,B).

**Figure 4:**
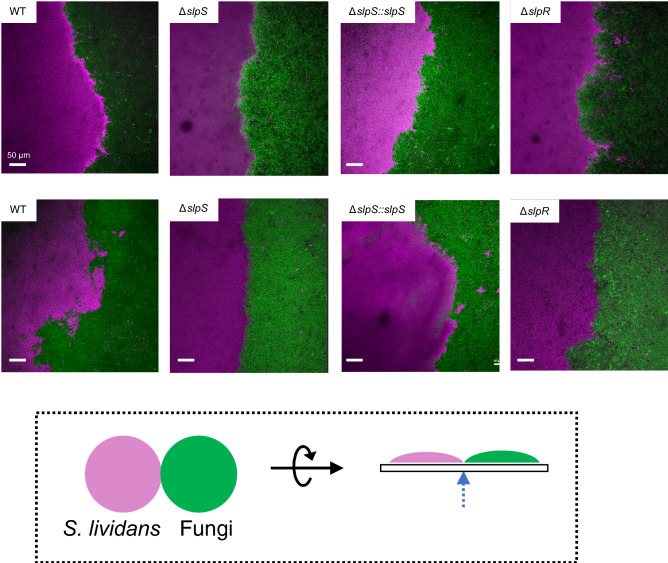
Confocal laser scanning microscopy (CLSM) of the colony boundaries between *S. lividans* and fungi. Colony boundaries between *S. lividans* and fungi were observed by CLSM. (**A**) *S. lividans* strains were co-cultured with *S. pombe* (top) and *S. cerevisiae* (bottom) on a cellophane, and the bottom of the colony boundary was then observed by CLSM. A dotted arrow in the bottom panel indicates the observed region between the colonies. *S. lividans* and fungi were visualized using Syto59 dye staining (magenta) and GFP expression (green), respectively. Scale bars, 50 μm.

To gain more insights into the competition between *S. lividans* and fungi, we performed confocal laser scanning microscopy (CLSM) to visualize the colony boundaries. *S. lividans* and the fungi were visualized using Syto59 dye and GFP, respectively. The CLSM revealed that the colony boundary between the TK23 strain of *S. lividans* and *S. pombe* tends to be distorted, whereas distinct borders are formed between the Δ*slpS* mutant and the fungi (Fig. 4). In addition, the *slpS*-complemented strain showed similar phenotypes to the parental strain (Fig. 4). These results show that the outcomes of the microbial competitions between *S. lividans* and the fungi can be altered by the presence or absence of SLP genes.

### Differential expression patterns of SLP genes and antibiotic biosynthesis genes under co-culture conditions

**Figure 5:**
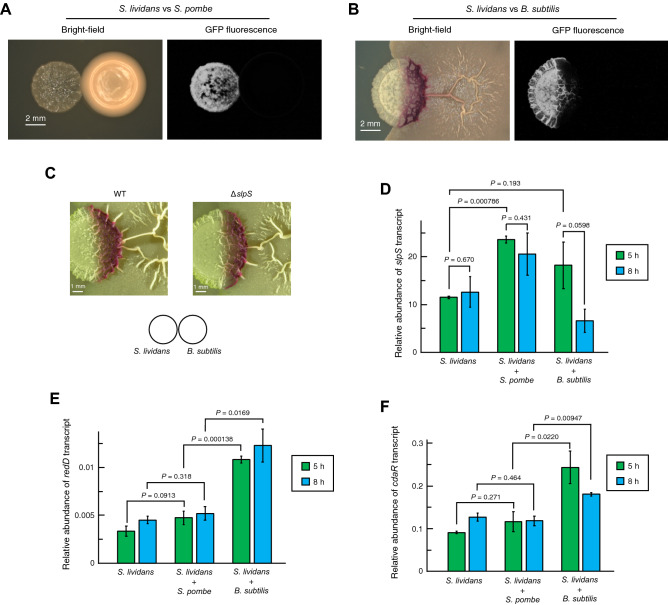
Patterns of SLP expression and antibiotic production by *S. lividans* in response to microbial competitions. Microscopic and transcriptional analysis revealed the differential response of *S. lividans* to the competitive interactions. *S. lividans* expressing SlpS-msfGFP was co-cultured with (**A**) *S. pombe* and (**B**) *B. subtilis* 168. Scale bars, 2 mm. (**C**) Colony boundaries between *B. subtilis* and *S. lividans* (TK23 strain and Δ*slpS* mutant) were observed. Scale bars, 1 mm. Relative transcription levels of (**D**) *slpS*, (**E**) *redD*, and (**F**) *cdaR* were compared among the different culture conditions. The amounts of the transcripts were normalized to the transcription level of *hrdB* encoding RNA polymerase principal sigma factor B. All values and the bars represent the mean value ± S.D. for three independent cultures. *P* values were calculated by *t* test with Welch's correction.

To further confirm the involvement of SLPs in the interkingdom competition, we analyzed SLP expression in *S. lividans* under co-culture conditions with fungal competitors. In the co-culture condition with *S. pombe*, SlpS-msfGFP fluorescence was clearly observed at the colony boundary between *S. lividans* and the fungi (Fig. 5A).

Furthermore, for simultaneous observation of the CLSM findings of the colony boundary and SlpS expression, we constructed an *S. lividans* strain expressing SlpS-mScarletI, the fluorescence of which can be detected separately from that of the Syto59 dye and GFP. When this *S. lividans* strain was cultured with fungal competitors, the fluorescence of SlpS-mScarletI was detected at the colony boundary (Supplementary Fig. 4), clearly indicating that *S. lividans* expresses SLPs in the contact region between these microorganisms.

We were also interested in whether SLPs are expressed at the colony boundary between *S. lividans* and the bacterial competitor such as *B. subtilis* the competition with which were not altered by the presence or absence of SLPs in our experiment. The microscopic observation of the expression of the fluorescent protein-labelled SlpS under the co-culture condition with *B. subtilis* 168 revealed that SlpS-msfGFP fluorescence was absent at the colony boundary, whereas *S. lividans* accumulated red pigments at the colony boundary, which strongly indicates the co-culture-dependent induction of secondary metabolite production, including that of the red antibiotic prodiginines (RED)^29–31^ (Fig. 5B). In the competition assay with *B. subtilis*, the Δ*slpS* mutant of *S. lividans* showed similar RED production to that of the TK23 strain, suggesting that the absence of SLPs at the colony boundary does not affect RED production induced by the interbacterial competition (Fig. 5C).

Given these results, we speculated that *S. lividans* increases antibiotic production in response to the interbacterial competition while decreasing SLP expression. We thus compared the transcription levels of the structural gene of SLP and antibiotic biosynthetic genes under co-culture conditions with bacterial or fungal competitors. We co-cultured *S. lividans* with either *S. pombe* or *B. subtilis* and extracted total RNA to quantify the amount of the transcripts of *slpS* and *redD*, a gene encoding a pathway-specific activator protein for RED production in *S. lividans*^32^. Subsequent quantitative reverse transcription polymerase reaction (RT-qPCR) analysis revealed that the transcription level of *slpS* was approximately two times higher in the co-culture with *S. pombe* than in the single culture (Fig. 5D), indicating that SLP expression was induced in response to contact with the fungus, whereas the *redD* transcription levels were comparable between these culture conditions (Fig. 5E). In contrast to the co-culture conditions with the fungal competitor, the transcription level of *slpS* was rapidly decreased during co-culture with *B. subtilis*, which is consistent with the absence of SlpS-msfGFP fluorescence at the colony boundary between *S. lividans* and *B. subtilis* (Fig. 5B). In addition, significantly higher levels of *redD* transcription in the co-culture with *B. subtilis* (Fig. 5E) support the previous observation that the induction of RED production in *S. lividans* can be triggered by interbacterial competition (Fig. 5B)^29–31^.

Besides RED, *S. lividans* produces several antibiotics such as calcium-dependent antibiotic (CDA), a channel-forming antibiotic that is effective against various Gram-positive bacteria, including *B. subtilis*^33,34^. CDA production is regulated by *cdaR* encoding a pathway-specific activator protein CdaR^35^. The transcription levels of *cdaR* under the co-culture condition with *B. subtilis* showed similar tendency to that of *redD* (Fig. 5F), indicating that the co-culture with *B. subtilis* also triggered the upregulation of the biosynthetic genes responsible for CDA production. Moreover, although co-culture with *B. subtilis* showed these positive effects on the antibiotic production in *S. lividans*, co-culture with *S. pombe* did not alter the transcription level of *cdaR* (Fig. 5F). In addition, this tendency of the transcriptional responses of *S. lividans* to *S. pombe* was also observed in the co-culture with *S. cerevisiae* (Supplementary Fig. 5). Together with the results of the microscopic analysis (Fig. 5A,B), these transcriptional responses demonstrate that the patterns of SLP expression and antibiotic production in *S. lividans* can differ depending on its competitor species.

## Discussion

In recent years, bioinformatic analyses have revealed that eCIS gene clusters are highly conserved among diverse bacterial classes, including Gram-negative and Gram-positive bacteria and archaea^8,9^. To date, the biological significance of eCIS has been established in only a few Gram-negative bacterial species. Anti-feeding prophage, an intensively studied model of eCIS, was discovered as a plasmid-encoded phage tail-like contractile injection system from *Serratia entomophila*, a causal agent of amber disease of the New Zealand grass grub *Costelytra zealandica*^13^. Further studies have revealed that anti-feeding prophage encoded by a gene cluster comprising 18 open reading frames is a key factor for eliciting amber disease in grass grub^13^. *Photorhabdus* virulence cassettes, produced by the symbiotic *Photorhabdus* species, are another model of eCIS with insecticidal activity^14^ and are chromosomally encoded in a pathogenicity island in which a diverse repertoire of virulence genes combating the hosts is encoded^14^. Conversely, metamorphosis-associated contractile structures from *Pseudoalteromonas luteoviolacea* represent eCISs with distinct structural properties and biological functions^15^. Metamorphosis-associated contractile structures were isolated from *P. luteoviolacea*, which exists in a symbiotic partnership with a marine tubeworm, and shown to be essential in the invertebrate’s metamorphosis^15^. Although these previous observations have demonstrated the versatile functions of eCISs in the interkingdom interactions between prokaryotes and eukaryotes, to the best of our knowledge, there has been no report elucidating the biological function of eCISs conserved in Gram-positive bacteria. One of the largest groups in which eCIS-related genes are widely conserved is Gram-positive actinomycetes, especially the *Streptomyces* species^8,9^. Since the first description by Kim et al*.* in 2005^19^, it has been strongly suggested that eCIS-related genes confer selective advantages to *Streptomyces* species^9,18,19^. The importance of these eCIS-related genes of *Streptomyces* is also suggested by the fact that they are often encoded in the central region of their linear genomes where many of the essential genes are encoded^26^. However, the biological significance of these genes has so far remained unclear because of the absence of apparent phenotypic changes in the deletion mutants for these genes under standard laboratory conditions^18,19^. In the present study, we have shown that SLPs, which are eCIS-like nanostructures produced by *Streptomyces*, could confer ecological benefits to *S. lividans* in microbial competitions. Our results indicate that SLP production protects *S. lividans* colonies from fungal invasion.

*Streptomyces* species and fungi are ubiquitously found in polymicrobial communities such as soil, occupying a substantial niche within the densely populated environments^28^. In such environments wherein nutrient availability is often limited^1,2^, SLP-mediated defense against fungal invasion could be beneficial to *Streptomyces* species because the defensive role of producing SLPs may increase the possibility of *Streptomyces* species acquiring nutrients, forming spores, and ultimately occupying the ecological niches. Another example of recently reported competitive interactions between *Streptomyces* species and fungal competitors is an indirect interkingdom interaction that triggered the morphological transition of *S. venezuelae*^36^. In this interaction, glucose depletion by neighboring fungi triggered the exploratory growth of *S. venezuelae*; the *S. venezuelae* colony rapidly traversed both the biotic and abiotic surfaces^36^. Although this exploratory phenotype was observed in some *Streptomyces* isolates, it is notable that most of the model *Streptomyces* species, including *S. lividans* and *S. coelicolor*, do not exhibit the exploratory phenotype^36^. In addition, the transcription levels of the SLP-related genes conserved in *S. venezuelae* remain unchanged in the phenotypic transition induced by glucose limitation, indicating that SLP-related genes are not involved in the exploratory growth of *S. venezuelae*^36^. Therefore, this distinct mode of growth would be irrelevant to the SLP-mediated competition between the *Streptomyces* species and fungi that we found in this study. Although an underlying mechanism of SLP-mediated microbial interactions is under investigation, our findings would provide new insights into interkingdom competitions that occur between *Streptomyces* species and its eukaryotic competitors. Further, the constitutive SLP expression in the absence of the fungal competitors suggests that the expression of SLPs can be triggered in response to, in addition to the interaction with the fungal competitors, general environmental stimuli such as nutrient levels which could provoke *bldA*-dependent gene expressions^17^. In this view, producing SLP may be connected to adaptation to various environmental conditions and may thus have biological significance in a broad range of growth conditions besides the microbial competition. Applying growth conditions that closely mimic the natural settings of *Streptomyces* would facilitate further clarification of the biological roles of SLPs, and this will be our next challenge.

It has been established that competitive interactions between *Streptomyces* species and other bacteria could be mediated by antibiotics^4,5,29,37^. This has been exemplified by a co-culture model system of *S. lividans* and *B. subtilis*, in which *S. lividans* accumulates antibiotic RED upon contact with *B. subtilis* colonies, whereas *B. subtilis* produces bacillaene, which exhibits growth inhibitory activity against *Streptomyces* species^4,29^. In the present study, we found that SLP expression in *S. lividans* was decreased during co-culture with *B. subtilis*, whereas antibiotic production was significantly increased as previously reported^4,29^ (Fig. 5). Similar transcriptional responses in interbacterial competitions have been reported in the previous study; the study demonstrated that SLP gene expression is decreased in *S. coelicolor* under co-culture conditions with the bacterial competitor *Myxococcus xanthus*, whereas the expression of genes involved in antibiotic production was elevated^5^. These observations and our results suggest that the upregulation of the genes responsible for antibiotic production and eventual decrease of SLP expression may be common responses between these *Streptomyces* species and the prokaryotic competitors. However, it should be noted that the transcription level of *slpS* under co-culture conditions with *B. subtilis* was initially increased and then dramatically decreased within 3 h (Fig. 5D). This suggests that the initial expression of SLPs might be triggered by a mechanism involving a general response to competitor microorganisms, including both bacteria and fungi, although knowledge on such mechanisms is currently limited in *Streptomyces* species^30,31^. In addition, metabolic switching from SLP expression, which is likely to burden *S. lividans* by consuming considerable resources and energy (Supplementary Figs. 2 and 3)^21^, to antibiotic production might have occurred in the case of the competition with *B. subtilis* and possibly other bacterial competitors^4,5,29^. These implications of the recognition of other microorganisms by *Streptomyces* species and the differential responses to the microbial competitions would raise interesting questions regarding the behavior of *Streptomyces* species in nature.

In the present study, we demonstrate that *Streptomyces* species produce SLPs, that can potentially protect *Streptomyces* colonies from fungal invasion. Differential patterns of SLP expression and antibiotic production by *Streptomyces* species in response to microbial competition are suggestive of as-yet unidentified strategies of *Streptomyces* species to survive and thrive in diverse environments densely populated with various microorganisms. Further, our findings also provide insights into co-culture-based approaches aiming at the discovery of the novel traits of *Streptomyces* species, including the production of new antibiotics.

## Methods

### Strains and culture conditions

The strains used in this study are listed in Supplementary Table 1. *Streptomyces* species were cultured in Bennett's glucose (BeG) medium comprising 0.1% (w/v) yeast extract, 0.1% (w/v) meat extract, 0.2% (w/v) NZ amine, and 1% (w/v) glucose. The pH was adjusted to 7.2. For measurement of the growth curve, 10^5^ viable spores were inoculated in 100 mL of liquid BeG medium and incubated at 30 °C with shaking at 180 rpm. The *Streptomyces* species and fungi were co-cultured in yeast extract with supplements (YES) medium comprising 0.5% (w/v) and 1% (w/v) glucose. The co-culture experiment was performed as follows: *Streptomyces* (10^4^ viable spores) were inoculated onto solid YES medium and cultured at 30 °C for 20 h. The fungi were precultured in 10 mL liquid YES medium for 1 day and then inoculated (10 μL) onto a site adjacent to the precultured *S. lividans* colony. For co-culture with *B. subtilis*, solid BeG medium was used. The initial distance between the colonies was approximately 1–2 mm. The cultivation periods varied from 4 to 7 days depending on the co-culture conditions. If required, *S. lividans* and fungi were co-cultured on a cellophane placed onto a solid medium.

### Genetic manipulations

Gene disruption of *Streptomyces* species was performed as follows. For *S. lividans*, the pK18mob plasmid was used as a conjugation vector; it was digested with EcoRI and HindIII and then fused with the amplified flanking regions (approximately 2 kbp each) of a gene of interest using In-Fusion (Clontech Laboratories Inc., CA, USA). The constructed plasmid was transformed into *E. coli* S17-1. The plasmid was then transferred from the transformed *E. coli* S17-1 to *S. lividans* by conjugation. Gene-disrupted mutants were obtained through homologous recombination events and consequent in-frame deletion of the targeted gene. Kanamycin (20 μg/mL) was used as a selection marker. For fusion of *slpS* with fluorescent proteins, the C-terminal region of *slpS* was amplified and fused with a flexible linker and codon-optimized *msfgfp* or *mscarletI*. The fused sequence was then introduced into the pK18mob plasmid, and the resultant plasmid was transferred to *S. lividans* as described above. Replacement of native *slpS* with *slpS-msfgfp* or *slpS-mscarletI* was completed through homologous recombination events. For gene disruption in *S. albus*, a pAT19 plasmid carrying an apramycin resistance cassette was used instead of pK18mob. *E. coli* ET12567 (pUB307) was used as a donor strain for conjugation. Each of the internal sequences of *XNR_0535* and *XNR_0530* was cloned into the pAT19 plasmid and then transferred to *S. albus*. Each gene was disrupted by a single homologous recombination event. The resultant mutants were obtained using apramycin (20 μg/mL) as a selection marker. Primers and plasmids used in this study are listed in Supplementary Table 2.

### Extraction of SLPs

SLPs were extracted from *Streptomyces* mycelia as follows. Viable spores (10^5^ per plate) were spread onto a cellophane placed on a BeG agar plate. After incubation at 30 °C for 3–5 days, the mycelia were scraped and resuspended in lysis buffer containing 50 mM Tris–HCl buffer (pH 7.5), 150 mM NaCl, 1 mg/mL lysozyme, 100 μg/mL DNase, 1% (v/v) Triton X-100, and a protease inhibitor cocktail. The resuspended solution was incubated at 37 °C for 2 h. After centrifugation at 15,000 × *g* for 10 min, the supernatant was ultracentrifuged at 200,000 × *g* for 60 min. The pellets were resuspended in resuspension buffer containing 50 mM Tris–HCl buffer (pH 7.5), 150 mM NaCl, and a protease inhibitor cocktail. The resultant solutions were subjected to further analysis. For TEM, the extracted SLPs were attached to thin carbon film-coated TEM grids (ALLIANCE Biosystems, Osaka, Japan) and washed with H_2_O. SLPs were then visualized via negative staining.

### Microscopic analysis

CLSM was performed using LSM880 equipped with objective LD LCI Plan-Apochromat 25 × /0.8 Imm Corr DIC M27 (Carl Zeiss, Oberkochen, Germany). The mycelia of *S. lividans* were visualized as follows. *S. lividans* and fungi were co-cultured on a cellophane placed on solid YES medium. After cultivation, the cellophane was transferred to fresh YES medium containing 5 μg/mL Syto59 and subjected to CLSM. Note that fungal cells appear not to be stained by Syto59 dye, probably due to robust transporters pumping out the dye. The colony was observed using Axio Zoom (Carl Zeiss).

### Bioinformatic analyses

The amino acid sequences of the eCIS tube proteins were obtained from database for eCIS^9^. Evolutionary analyses were conducted in MEGA X^38^. The tree was drawn to scale, with branch lengths in the same units of the evolutionary distances used to infer the phylogenetic tree. All ambiguous positions were removed for each sequence pair (pairwise deletion option). Bootstrap values were calculated from 500 replicates. Comparison and visualization of gene cluster similarity were performed using clinker, a Python-based tool, and clustermap.js, a companion JavaScript visualization library (https://github.com/gamcil/clinker)^39^.

### RT-qPCR analysis

Transcriptional analysis of *S. lividans* was performed as follows. *S. lividans*, *B. subtilis*, and *S. pombe* were precultured in 10 mL liquid YES medium for 20 (*B. subtilis*) or 42 (*S. lividans*, *S. pombe* and *S. cerevisiae*) h. The precultured cells were then washed and resuspended in fresh YES medium. An equivalent volume (500 μL) of the resuspended solutions were mixed in sterilized 1.5-mL tubes and incubated at 30 °C. To note, wet weights of the inoculated cells were adjusted to 30–50 mg/tube for all culture conditions. After incubation, the tubes were centrifuged at 7000 × *g* for 10 min. Total RNA was extracted from the harvested cells using the RNeasy Mini kit (QIAGEN, Venlo, Netherlands).

RT-qPCR was performed as follows. The extracted RNA was treated with DNase and then purified via ethanol precipitation. The purified RNA was subjected to cDNA synthesis. The synthesized cDNA was then mixed with TB Green master mix (Takara Bio Inc., Shiga, Japan) and primers (0.2 μM each) just before the subsequent reaction. Reaction specificities were confirmed using melting curve analysis. The thermal profile of the qPCR was as follows (2-step PCR protocol): 95 °C for 3 min, 95 °C for 5 s, and 60 °C for 30 s (30 cycles). The standard curve for the calculation of relative abundance of each transcript was obtained using sequentially diluted genomic DNA (0.05–50 ng) as templates. Primers used are listed in Supplementary Table 2.

## Supplementary Information

Supplementary Information.

## Data Availability

The datasets supporting the current study are available from the corresponding author on request.
