# Supplementary material for: Phage tail-like nanostructures affect microbial interactions between Streptomyces and fungi
Source: Sci Rep. 2021 Oct 11;11:20116. doi: 10.1038/s41598-021-99490-8 (PMC8505568; doi:10.1038/s41598-021-99490-8)
Supplement: Supplementary file 1 — Supplementary Information. [file 41598_2021_99490_MOESM1_ESM.docx]

Supplementary Information

Phage tail-like nanostructures affect microbial interactions between *Streptomyces* and fungi

Toshiki Nagakubo^1,*^, Tatsuya Yamamoto^2^, Shumpei Asamizu^1,3^, Masanori Toyofuku^2,4,5^, Nobuhiko Nomura^2,4^, Hiroyasu Onaka^1,3,*^

^1^ Graduate School of Agricultural and Life Sciences, Department of Biotechnology, The University of Tokyo, Tokyo, Japan

^2^ Department of Life and Environmental Sciences, University of Tsukuba, Tsukuba, Japan.

^3^ Collaborative Research Institute for Innovative Microbiology, The University of Tokyo, Tokyo, Japan

^4^ Microbiology Research Center for Sustainability (MiCS), University of Tsukuba, Tsukuba, Japan

^5^ Suntory Rising Stars Encouragement Program in Life Sciences (SunRiSE) Fellow

*Corresponding Authors: Toshiki Nagakubo and Hiroyasu Onaka

nagakubo@g.ecc.u-tokyo.ac.jp

aonaka@g.ecc.u-tokyo.ac.jp

**Supplementary Figure 1. Transmission electron microscopy analysis of the mycelial extracts of *Streptomyces* strains.**

Deletion mutants for SLP-related genes do not produce phage tail-like particles. Each strain derived from *S. lividans* TK23 (Δ*slpS*, Δ*slpR*, Δ*slpS*::*slpS* and Δ*bldA*) and *S. albus* J1074 (Δ*XNR_0535* and Δ*XNR_0530*) was grown on a solid medium and its mycelia were subjected to the extraction and microscopic analysis as described under Methods. Yellow arrowheads indicate SLPs produced by *slpS*-complemented strain of *S. lividans*. Scale bars, 100 nm.

**Supplementary Figure 2. Deletion of *slpR* affects the growth rate of *Streptomyces lividans*.**

Growth curves of *S. lividans* strains are shown. In-frame deletion mutants for *slpS* (a SLP sheath protein) and *slpR* (a transcriptional regulator for SLP gene cluster) were constructed as described under Methods. These mutants and the parental strain were grown in liquid BeG medium. At each time point, mycelia were harvested from 1 mL of culture and then dried cell weights were measured. All values and the bars represent the mean value ± S.D. for three independent cultures.

**Supplementary Figure 3. SlpS-msfGFP expression in *Streptomyces lividans* grown on solid medium.**

*S. lividans* strains were grown on solid BeG medium and observed. GFP fluorescence was displayed in gray-scale. Scale bars, 200 μm.

**Supplementary Figure 4. Distribution of SlpS-mScarletI at the colony boundaries between *Streptomyces lividans* and fungi.**

SlpS expression in *S. lividans* was observed under co-culture conditions. *S. lividans* mycelia and fungal cells were visualized by Syto59 dye (magenta) and GFP (green), respectively. Distribution of SlpS at the colony boundaries were visualized by detecting SlpS-mScarletI fluorescence (cyan).

**Supplementary Figure 5. Transcriptional analysis of *S. lividans* under the co-culture condition with *S. cerevisiae*.**

Relative transcription levels of *slpS*, *redD*, and *cdaR* under the co-culture condition with *S. cerevisiae* were analyzed by RT-qPCR. *S. lividans* was cultured with or without the fungal competitor (*S. cerevisiae*) and the amounts of the gene transcripts were quantified as described under Methods. These values were normalized to the transcription level of *hrdB* encoding RNA polymerase principal sigma factor B. Finally, the transcription levels of the target genes under the co-culture condition were normalized to those under the single culture condition. All values and the bars in this figure represent the mean value ± S.D. for three independent cultures.

**Supplementary Table 1. Microorganisms used in this study.**

**Supplementary Table 2. Primers and plasmids used in this study.**
